# Effectiveness of a Multicomponent Intervention in Primary Care That Addresses Patients with Diabetes Mellitus with Two or More Unhealthy Habits, Such as Diet, Physical Activity or Smoking: Multicenter Randomized Cluster Trial (EIRA Study)

**DOI:** 10.3390/ijerph18115788

**Published:** 2021-05-28

**Authors:** Francisco Represas-Carrera, Sabela Couso-Viana, Fátima Méndez-López, Bárbara Masluk, Rosa Magallón-Botaya, Jose I. Recio-Rodríguez, Haizea Pombo, Alfonso Leiva-Rus, Montserrat Gil-Girbau, Emma Motrico, Ruth Martí-Lluch, Francisco Gude, Ana Clavería

**Affiliations:** 1 Galicia South Health Research Institute, Hospital Álvaro Cunqueiro, Technical Block, Floor 2, Roal Clara Campoamor nº 341, 36213 Vigo, Spain; sabela.couso@iisgaliciasur.es (S.C.-V.); anaclaveriaf@gmail.com (A.C.); 2 Aragonese Research Group in Primary Care (GAIAP), Institute of Health Research, Avenue San Juan Bosco, 13, 50009 Zaragoza, Spain; fatima.lopezmendez@hotmail.com (F.M.-L.); bmasluk@unizar.es (B.M.); med000764@gmail.com (R.M.-B.); 3 San Juan Health Centre, Salamanca Primary Care Research Unit (APISAL), Institute of Biomedical Research of Salamanca (IBSAL), Department of Nursing and Physiotherapy (University of Salamanca), Avenue Portugal 83, 2 Floor, 37005 Salamanca, Spain; donrecio@gmail.com; 4 Ezkerraldea-Enkarterri-Cruces Integrated Health Organisation, Biocruces Bizkaia Health Research Institute Innovation Unit, Plaza de Cruces s/n, 48903 Barakaldo, Spain; haizea.pomboramos@osakidetza.eus; 5 Balearic Islands Health Research Institute (IdISBa), Highway Valldemosa 79, 07120 Palma, Spain; aleiva@ibsalut.caib.es; 6 Research Group in Health Technology Assessment in Primary Care and Mental Health (PRISMA), Research and Development Unit, Institut de Recerca Sant Joan de Déu, Parc Sanitari Sant Joan de Déu, Street Dr. Antoni Pujadas, 42, 08830 Sant Boi de Llobregat, Spain; m.gil@pssjd.org; 7 Psychology Department, Universidad Loyola Andalucía, Avenue of the Universities, s/n, 41704 Dos Hermanas, Spain; emotrico@uloyola.es; 8 Girona Research Support Unit, Jordi Gol i Gurina University Institute for Research in Primary Health Care Foundation (IDIAPJGol), Street Maluquer Salvador 11, 17002 Girona, Spain; rmarti.girona.ics@gencat.cat; 9 Clinical Epidemiology Unit, Research Methods Group, Santiago Institute of Sanitary Research (IDIS), Complejo Hospitalario Universitario de Santiago, Travesía da Choupana, s/n, 157056 Santiago de Compostela, Spain; francisco.gude.sampedro@sergas.es

**Keywords:** health promotion, diabetes mellitus, exercise, Mediterranean diet, tobacco use disorder, primary health care

## Abstract

*Introduction:* We evaluated the effectiveness of an individual, group and community intervention to improve the glycemic control of patients with diabetes mellitus aged 45–75 years with two or three unhealthy life habits. As secondary endpoints, we evaluated the inverventions’ effectiveness on adhering to Mediterranean diet, physical activity, sedentary lifestyle, smoking and quality of life. *Method:* A randomized clinical cluster (health centers) trial with two parallel groups in Spain from January 2016 to December 2019 was used. Patients with diabetes mellitus aged 45–75 years with two unhealthy life habits or more (smoking, not adhering to Mediterranean diet or little physical activity) participated. Centers were randomly assigned. The sample size was estimated to be 420 people for the main outcome variable. Educational intervention was done to improve adherence to Mediterranean diet, physical activity and smoking cessation by individual, group and community interventions for 12 months. Controls received the usual health care. The outcome variables were: HbA1c (main), the Mediterranean diet adherence score (MEDAS), the international diet quality index (DQI-I), the international physical activity questionnaire (IPAQ), sedentary lifestyle, smoking ≥1 cigarette/day and the EuroQuol questionnaire (EVA-EuroQol5D5L). *Results:* In total, 13 control centers (*n* = 356) and 12 intervention centers (*n* = 338) were included with similar baseline conditions. An analysis for intention-to-treat was done by applying multilevel mixed models fitted by basal values and the health center: the HbA1c adjusted mean difference = −0.09 (95% CI: −0.29–0.10), the DQI-I adjusted mean difference = 0.25 (95% CI: −0.32–0.82), the MEDAS adjusted mean difference = 0.45 (95% CI: 0.01–0.89), moderate/high physical activity OR = 1.09 (95% CI: 0.64–1.86), not living a sedentary lifestyle OR = 0.97 (95% CI: 0.55–1.73), no smoking OR = 0.61 (95% CI: 0.54–1.06), EVA adjusted mean difference = −1.26 (95% CI: −4.98–2.45). *Conclusions:* No statistically significant changes were found for either glycemic control or physical activity, sedentary lifestyle, smoking and quality of life. The multicomponent individual, group and community interventions only showed a statistically significant improvement in adhering to Mediterranean diet. Such innovative interventions need further research to demonstrate their effectiveness in patients with poor glycemic control.

## 1. Introduction

Diabetes mellitus (DM) is a world public health problem that affects 60 million people in Europe. Different health care organizations face this disease, which has a strong added socioeconomic impact [1]. This challenge not only centers on caring for DM itself, but also on its associated complications, with increasingly higher morbidity-mortality rates [2].

Healthy life habits directly influence glycemic control, which is why they are related to DM diagnosis, treatment and prognosis [1,2]. Every percentage of lowering HbA1c means 43% less peripheral artery disease, 37% fewer microvascular complications, 21% lower DM-related mortality, 14% fewer cardiac infarctions and 12% fewer strokes [3]. Handling this complex disease also affects people’s quality of life, and DM patients’ quality of life tends to be worse than for people without chronic diseases [4]. Finally, the worse their quality of life, the more difficult it is to control HbA1c [5].

Most adults present several unhealthy life habits that are interrelated [6]. Galán et al. [7] state that 20% of the Spanish population adopts three or four unhealthy life habits, and smoking is the factor that mostly frequently interrelates to the others.

The Spanish population smokes more than average in the European Union [8] despite smokers and passive smokers being at higher risk of cardiovascular disease, premature death and microvascular complications [9,10]. In type 2 DM, smoking cessation entails significant benefits to help HbA1c to lower [11].

Physical exercise considerably improves HbA1c regardless of weight loss [12,13]. Recent studies have related living a sedentary lifestyle to a higher risk of premature death independently of the level of physical activity practiced [14]. In DM, avoiding a sedentary lifestyle also helps glycemic control [15].

Eating a Mediterranean diet substantially lowers HbA1c, delays having to take antidiabetics and reduces cardiovascular events and overall mortality [16,17,18]. Given the Spanish population’s Mediterranean nature, food recommendations must be supported by this diet pattern [19].

As primary care (PC) interventions to change unhealthy life habits have increased in recent years, studies to evaluate their effectiveness are needed [6,20]. In practice, the most widespread strategy to bring about change in conduct is to advise about a single unhealthy habit or risk factor [21]. Nevertheless, this approach type has a weaker impact on people’s health compared to interventions that address several unhealthy life habits (multi-risk approach) [22]. Another question lies in the way these life habits are dealt with and, although individual and group approaches are effective, they are normally implemented separately [23,24].

The combination of several components in a single intervention is what defines “multicomponent interventions” [25]. Research into the effectiveness of such interventions is ample and varied and has analyzed several approaches according to the type of intervention and methodology used to promote health. With DM, these kind of interventions performed to date are not backed by solid evidence for simultaneously dealing with different unhealthy life habits [26,27].

Therefore, the multicomponent intervention in the present study was conducted in PC by focusing on diet, physical activity and smoking to include individual, group and community components. These three types of approaches are essentials in any organized diabetes-based education program to suitably achieve disease self-management [1].

In 2012, the Research Network in Preventive and Health Promotion Activities (redIAPP) [28] started designing a multicomponent intervention in Spain for people aged 45–75 years to promote healthy life habits to improve quality of life and avoid high-prevalence chronic diseases. This project was called EIRA. To date, the results of the first three phases (preclinical, I and II) have been published [29,30,31,32]. This article presents the results of the patients with DM included in the EIRA trial [33]. A hypothesis was posed: the multicomponent intervention would find statistically significant differences in the glycemic control of DM patients with several unhealthy life habits compared to those receiving the usual PC.

The main objective of this study was to assess the effectiveness of a multicomponent intervention in the glycemic control of patients with DM aged 45–75 years with two unhealthy life habits or more (smokers, not practicing enough physical activity and/or barely adhering to Mediterranean diet). Its secondary objective was to assess the effectiveness of this intervention in adhering to Mediterranean diet, physical activity, sedentary lifestyle, smoking and quality of life.

## 2. Materials and Methods

### 2.1. Design

A randomized clinical cluster (primary health care centers) trial with a control group was used. This randomized clinical trial (RCT) is known as the EIRA trial, which is registered at ClinicalTrials.gov with number “NCT03136211”.

The article presents the results of an analysis performed with all the patients diagnosed with DM who were included in the EIRA trial [33]. Consolidated Standards of Reporting Trials (CONSORT) recommendations were followed to present the results [34]. A cluster-randomized clinical trial (RCT) conducted with the subgroup of patients with DM was included in EIRA project phase 3 [19]. The trial registration number is NCT03136211. CONSORT recommendations were followed to present the results [20].

### 2.2. Study Area

This RCT was performed in 26 primary health care centers (PHCCs) in 7 health departments in Spain from January 2016 to December 2019. Spain’s national health system (NHS) guarantees health services in each Spanish territory/Spanish autonomous community by means of health departments. Health care is organized at two levels: PC and hospital care. PHCCs are made up of a multidisciplinary team with doctors, nurses, pediatricians, midwives, social workers, pharmacists and dentists who perform health care services, health education, health promotion/prevention and community activities.

### 2.3. Inclusion and Exclusion Criteria

PHCCs: The selected centers had to meet these criteria: not be located in areas with much sociocultural diversity or in areas with considerable tourism, have access to the internet, offer the possibility of prescribing community activities, recommend community activities and have professionals available who are particularly committed to this study. Professionals’ participation was voluntary.

Patients: The DM patients from the EIRA trial aged 45–75 years with two unhealthy life habits or more (smoking, nor adhering to Mediterranean diet or not practicing much physical activity) were included. The exclusion criteria were as follows: have a serious illness, cognitive impairment or severe mental illness, not dependently able to perform basic activities of daily living, participating in a long-term home health care program, being treated for cancer or receiving end-of-life care. Those patients who did not plan to reside in the area while the intervention lasted were also excluded [33].

DM patients included anyone who met any of the following criteria [35]: having a registered medical background of DM being diagnosed when the intervention started or ended, presenting HbA1c ≥6.5% in the 6 months before the intervention began and being prescribed any antidiabetic when the intervention began.

### 2.4. Intervention (EIRA Study)

An educational intervention to promote health about Mediterranean food, physical activity and smoking lasted 12 months, took place in PHCCs and was personally administered by patients’ doctors and nurses.

The professionals who participated in this intervention were trained for more than 60 h to standardize the intervention.

The EIRA intervention consisted in a multicomponent approach for each life habit, at three levels (individual, group and community) according to each patient’s conduct change stage [36]. The individual intervention consisted in a short educational intervention during consultations and sending reminder text messages. Those people in the contemplative and preparation phase could also employ a mobile application to perform continuous activity toward their physical activity and diet. Group interventions consisted in organizing a workshop and focused only on physical activity and diet. Finally, the community intervention included recommending community resources to help to improve adhering to the Mediterranean diet and increasing physical activity (social prescription) [33].

While the intervention was underway, health care professionals attempted to adapt the different intervention components to each patient’s characteristics (resources, expectations, requirements, etc.). During the first visit, professionals agreed with patients on a specific approach and follow-up plan.

The individual intervention included 2–3 visits and the possibility of further reinforcing the intervention. Depending on the change phase each patient was in regarding their life habits, the following took place: (1) “a very brief intervention” with the objective to raise more awareness of the need to change life habits and support any changes or help to prevent possible relapses; (2) “a brief intervention” to establish a specific agreed plan to change behavior. Health care professionals performed this brief intervention by applying motivational techniques after they received 20 h of online training [37]. This intervention type was supported by an informative website, sending personalized texts and using mobile applications or other electronic devices (pedometers, smart watches, etc.). At the end of consultations, the professionals involved in all the individual interventions handed out to each patient the written support material as a reinforcement mechanism.

The brief intervention for Mediterranean diet included personalized recommendations about the changes that each patient had to include to help them adhere to this diet pattern according to the therapeutic objectives set out. This food pattern is generally characterized by eating plenty of plant food like fruit and vegetables, legumes, dried fruit and nuts, cereals and rice. In the diet included in the intervention, olive oil was the main source of fat. Moderate amounts of fish, seafood, poultry, dairy products and eggs were included. Small portions of red meat and a very small daily amount of wine with meals were indicated [19].

The brief intervention to deal with smoking included setting day D to stop smoking, offering pharmacological/conduct treatment, and follow-up visits after 7–15 days and 1 month after smoking cessation.

The brief intervention for physical activity was based on the health care professionals reaching a consensus with patients, an individualized physical activity plan including community resources or specific programmers to be done in PHCCs to increase the chances of performing physical activity.

The group intervention took place during a health education workshop, and focused on healthy diet and physical activity. It aimed to reinforce the recommendations made in the individual intervention, and to provide patterns that helped to adhere to Mediterranean diet and practice physical exercise. This workshop began after the first individual intervention visit. It included two sessions lasting 90–120 min each, performed by the health care professionals from PHCCs.

The community intervention consisted of recommending social activities [38] in the patient’s own neighborhood according to the detected risky life habits and accessibility to community resources, e.g., gyms, allotments, healthy walking, etc. The professionals from PHCCs previously identified the community’s health assets [39] and selected the most appropriate ones according to the detected unhealthy behaviors, accessibility and the possibility of referring participants.

All the details of interventions are specified in the study protocol [33]. No blinding was included in relation to either the patients in the two study groups or the health care professionals who undertook the IRA intervention.

### 2.5. Usual Health Care (the PHCC Control Group)

The participants from the PHCC control group received usual health care in accordance with the national recommendations and guidance. If a change in life habits was necessary, it was recommended when DM patients went to consultations with their family doctors or nurses, where they received some brief advice [40,41].

### 2.6. Measures and Data Collection

The main outcome variable was HbA1c (%). The secondary outcome variables included adhering to Mediterranean diet (MEDAS) [42], adhering well to the Mediterranean diet (MEDAS ≥9), diet quality (DQI-I) [43], physical activity (IPAQ per category: low < 600 MET/moderate-high) [44], sedentary lifestyle (sat down ≥6 h/day) [45], smoking (≥1 cigarette/day) and quality of life (the VAS scale of EuroQol-5D5L) [46].

In order to evaluate the effectiveness of the EIRA intervention, the initial (0 months) and final (12 months) data of the outcome variables were compared. This information was collected in PHCCs by means of an external support unit to not increase the work overload of these professionals involved in the intervention. These professionals received specific training in the measuring instruments and about the electronic data collection notebook, which was designed ad hoc by a group of experts from redIAPP [28].

Supplementary Material File S1 offers details about the independent variables.

### 2.7. Sample Size

In order to calculate the EIRA trial sample size, a ≥8% difference was taken of those people showing a positive change in one life habit or more of the three life habits between both study groups [33]. Moreover, 30% of losses through follow-up, a 5% alpha risk, a 20% beta risk and a 0.01 intragroup correlation were taken [47]. According to these data, a minimum of 140 participants were considered for each PHCC, with 3640 people in all and 1820 in each study group (control and intervention). The PASS software was employed to calculate the sample size. For the present study, all the DM patients present in the final EIRA trial sample were selected.

In parallel, the DM sample needed to detect a minimum 0.3% reduction in the HbA1c value in these patients after the intervention was estimated [48,49,50], and it was necessary to study at least 420 people. Calculations were done using the Gpower freeware by considering the calculated sample size for the EIRA trial (3640).

### 2.8. Randomization

The randomization unit was PHCCs. This process was centralized on the University Institute Foundation for Research in Primary Health Care Jordi Gol, Barcelona (Spain) using the statistical R software. For the seven participating Spanish autonomous communities (SAC), half the PHCCs were randomly assigned to the control group (CG) and the other half to the intervention group (IG). This gave 13 PHCCs in the CF and 13 others in the IG.

**Figure 1:**
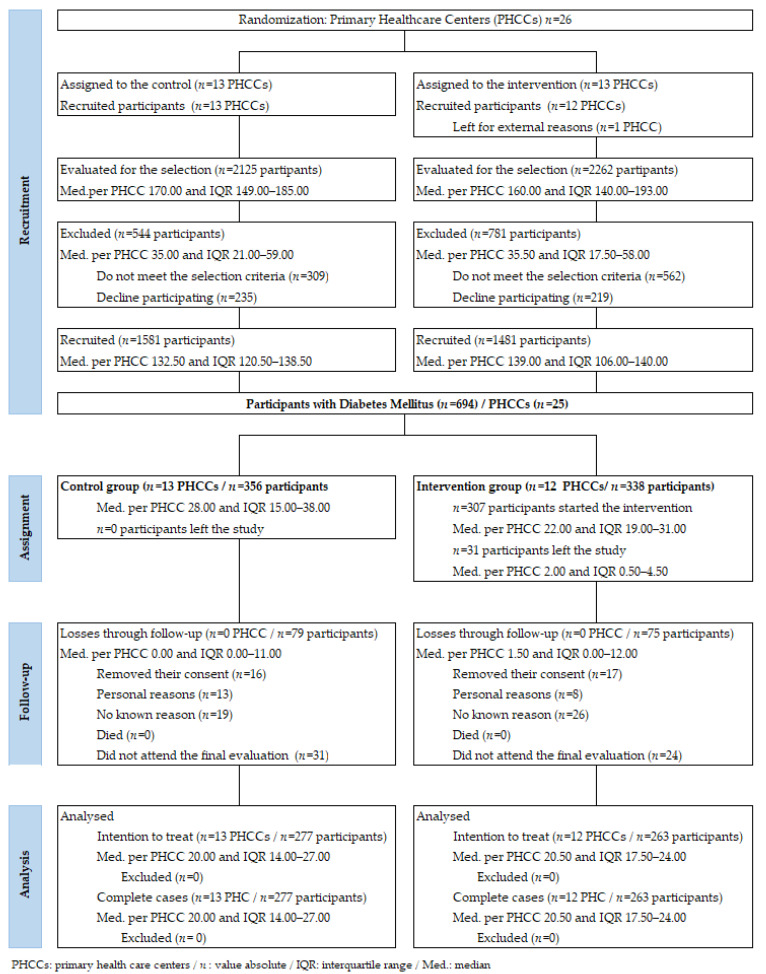
Flow diagram of the study.

Both the patients and professionals knew which group they had been assigned to. Figure 1 depicts the study’s flow diagram.

### 2.9. Recruitment

Population selection was performed opportunistically between February 2017 and January 2018 and was stratified by the age/gender distribution expected from the participating PHCCs.

The doctors and nurses from PHCCs performed population recruitment by the different methods set out in the study protocol [33]: (1) during the visits that form part of usual health care, (2) self-administered questionnaires handed out in waiting rooms or at admission desks, (3) advertising on posters inside PHCCs and (4) telephoning the selected patients by revising electronic medical records.

The professionals were previously trained to ensure a homogenous procedure.

### 2.10. Statistical Analysis

The statistical analysis was performed with packages lme4 [51] and mice [52] of the R freeware [53] and SPSS v19 was also used [54].

Following the CONSORT recommendations for non-pharmacological interventions [34], and to reduce any bias due to lack of data during the follow-up, an intention-to-treat (ITT) analysis was carried out.

After sorting and analyzing the quality of the obtained data, multiple imputation was performed by random forest to avoid the collinearity influence [55]. Lost values were assumed to be values missing at random [56]. The following were included: intervention (yes/no), PHCCs, all the initial/final outcome variables, stratification variables (age, gender) and auxiliary variables with losses below 30% and the possible influence on outcome (comorbidities, motivation, clinical variables). The imputation phase created many copies of datasets, and each one contained different estimations of missed values. Then, the imputed datasets were analyzed.

In order to check the changes observed from the beginning to the end of the study in each group and for every outcome variable (primary and secondary), mixed multilevel effects were regressed (linear for continuous results, logistic for dichotomic results). Each model included the basal values of the outcome variables because they were repeated measures. In practice, such a high correlation like 0.7 is quite feasible for the same variable measured at the baseline after processing, and the fit of this baseline is most important [57]. This value and the intervention were taken as fixed effects and PHCCs as random effects. All this was performed using the *lme4* package [51], which identifies the model’s parameters that optimize the restricted maximum likelihood criterion.

This was how a set of estimations of parameters and standard errors was obtained, which were combined according to the guidance of Rubin [56]. As a result of the combination, the adjusted difference in the means between groups for the continuous results and the odds ratio (OR) for the dichotomic outcome variables were calculated with 95% confidence intervals (95% CI) in both situations.

Then, the regression analysis of the multilevel mixed effects was repeated with the complete cases for all the outcome variables in a similar manner to that previously described.

A descriptive study was done of the variables included in both the CG and IG. The quantitative variables were expressed with their absolute value (N), mean and standard deviation (SD) or median (med.) and the interquartile range (IQR). The qualitative variables were expressed with their absolute value, percentage (%) and 95% CI. To make the basal comparison, the following were used in each particular case: the Chi-squared test or Fisher’s exact test for the qualitative variables; the Mann–Whitney U or Student’s t-test for the quantitative variables.

Statistical significance (*p*) was evaluated at the <0.05 level (two-tail).

## 3. Results

Of our study population, 96% were recruited between March and September 2017, and the rest until January 2018, and 90% of the patients were opportunistically recruited during their visits to their PC doctors or nurses. The remaining 10% were recruited by telephone using each professional’s list of patients, and by self-administered questionnaires that were voluntarily completed as they were available in PHCCs.

**Figure 2:**
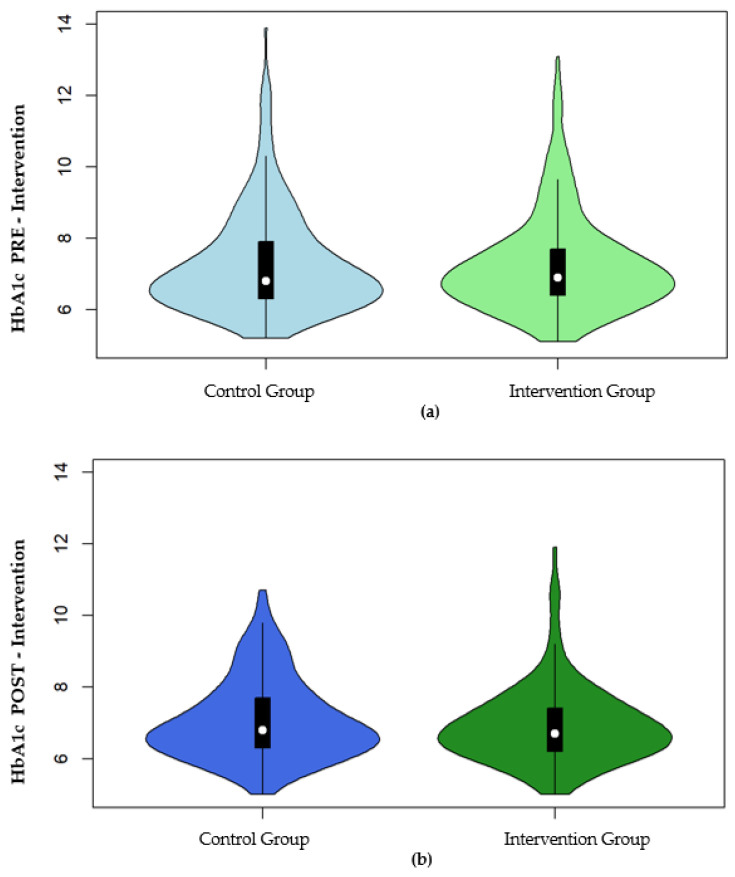
(**a**) HbA1c pre-intervention; (**b**) HbA1c post-intervention.

Figure 2 shows how one PHCC of the IG left the study for reasons beyond our control before the EIRA intervention began. This left 13 PHCCs for the CG and 12 for the IG.

We included 3062 patients, of whom 694 had DM (22.66% (95% CI: 21.22–24.18)) with 356 in the CG and 338 in the IG (*p* = 0.312): overall, 277 patients in the CG and 263 in the GI finished the study (Table 1). The PHCCs’ characteristics are provided in File S2 Table S1. The follow-up protocol is presented in File S2 Table S2.

The basal descriptive analysis of the study variables was published in the Revista Española de Salud Pública [58] and can be consulted in File S3.

According to Table 1, 62.36% (95% CI: 57.24–67.28) of the CG and 62.13% (95% CI: 56.87–67.18) of the IG were male, and the median of their age was 60 years for both groups with an IQR of 54–66 and 54–67, respectively. The analysis of the basal values showed no differences between both groups in most variables [58].

Table 2 presents the post-EIRA intervention descriptive analysis of the outcome variables.

The changes in HbA1c after the EIRA intervention can be graphically viewed in the violin graph (Figure 2).

Imputation and a multivariate analysis were carried out according to the protocol. Imputation firstly took place with five imputations on all five branches, and convergence was visually revised. Suitable regression models were selected for each outcome variable and outcomes were combined. The effectiveness of the intervention after the ITT analysis by applying the multilevel mixed models was adjusted by basal values and PHCCs and is found in Table 3. The complete cases analysis appears in File S2 Table S3.

The EIRA intervention only gave statistically significant results and showed a 0.45-point higher adherence to Mediterranean diet (95% CI: 0.01–0.89). The patients in the IG more correctly (1.62-fold) adhered to Mediterranean diet (95% CI: 1.03–2.54) than those in the CG.

To calculate its effect size, we converted standardized *β* weights (adjusted mean difference) from the multiple regression analysis to *r* [59], where *r* was 0.51, which is a large effect size.

The visual inspection of the model’s residue, identified for each outcome variable, revealed neither homoscedasticity nor deviations from normality.

## 4. Discussion

No statistically significant changes were found for either glycemic control or physical activity, nor for sedentary lifestyle, smoking and quality of life. The multicomponent intervention only showed a statistically significant improvement in adhering to the Mediterranean diet.

The main objective of the EIRA intervention was to evaluate the effectiveness in improving the DM population’s glycemic control. However, no statistically significant improvements were achieved in improving glycemic control, as measured by glycated hemoglobin, because it lowered by only 0.09%. Evidently, the better glycemic control is prior to intervention, the more difficult it is to achieve significant improvements [49]. In our study, half the patients’ glycemic control was below <8% before the intervention started. Nevertheless, we observed a higher probability of achieving glycemic control below 8% (OR = 0.57) compared to another similar study (OR = 0.20) that also performed an educational multicomponent intervention with DM patients with 7%–9% HbA1cf [60]. That study achieved significant post-intervention improvements, mostly because it began with higher former glycated hemoglobin figures which, therefore, allowed for a wider improvement margin. Likewise, a recent meta-analysis published in 2019 [61], which aimed to assess the effect of Mediterranean diet on improving glycated hemoglobin, demonstrated that it generally lowered by 1%. Adhering to Mediterranean diet for more than 12 months would very likely give lead to biochemical significance. Hence, there is a need to include interventions that provide evidence in day-to-day clinical practice. If not, they run the risk of fading away with time [62].

The main finding with the Mediterranean diet was demonstrating a statistically significant improvement in DM patients adhering to Mediterranean diet. Regarding the clinical impact of these results, the effect size obtained indicates that this is also a clinically relevant result, according to Cohen’s cut-offs in educational research [63].

The specific objective of MEDAS was to improvement shown by 0.45 points and the DQI index of 0.25 compared to the initial values. These improvements were also observed in a similar intervention for 12 months, but with a selected population of DM patients (1.7 and 1.3, respectively) [64]. Perhaps performing an intervention with a given population group covers more specific aspects of interest than interventions with bigger and more diverse population groups. This and other studies [65], including the present one, demonstrate that this type of intervention has a clinical impact on patients acquiring healthy eating habits. It can be applied to health services and presents excellent cost effectiveness because it is a simple intervention, PC staff are perfectly qualified to perform it and it incurs no additional costs.

The results obtained for physical activity were not statistically significant for the sedentary lifestyle and IPAQ scale values. For physical activity, lack of observed effectiveness matches the statement by Hamilton et al. about patients with diabetes being more reluctant to perform moderate/high physical activity compared to people with no chronic disease, and for whom DM patients tend to spend most of the day performing activities generally typical of a sedentary lifestyle [66].

In relation to smoking, despite the intervention managing to lower the number of smokers, the results were not statistically significant. Although most DM patients did not smoke before the intervention started, our findings coincide with those published in the review by Ebrahim et al., where multicomponent interventions did not show better smoking cessation success than classic approaches that are more individual and performed during consultations [67].

DM patients’ quality of life did not significantly improve after the intervention. However, the final evaluation was positive with an EVA of 70 (med.) out of 100. Compared to previous works in the literature, some studies report significant improvements in quality of life, but they applied interventions that specifically addressed a study population with type 2 DM [68].

As set out in the WHO’s “Global Strategy On Diet, Physical Activity and Health” [69] for diabetes, actions must center on population measures that promote healthy habits to lower this growing disease burden worldwide by taking different approaches (individual, group, community). This was our approach and, yet, several factors might have contributed to our results not being as effective as expected. A clinical trial [33] addresses individual, group and community interventions in patients with various risks or life habits. This means that our EIRA intervention was designed for people whose life habits could improve, and not specifically for patients with DM. The present study analyzed the effect of this multicomponent intervention on a single population group: patients with DM. Perhaps the general approach based on comorbidity and many life habits and patients with DM being selected in line with them made the intervention less effective for these patients than other more specific interventions in our setting [70,71] and in other different socio-cultural contexts [72,73], which could have impacted poorer results than the overall clinical trial sample overall.

The effectiveness of the IPAQ measuring instrument is acceptable when employed in population studies [74]. However, the literature warns about its limitations for measuring physical activity over long periods of time and recommends using accelerometry whenever possible [75]. However, this was not possible in our study for logistic reasons.

Other factors could explain the EIRA intervention’s lack of effectiveness overall. One such factor is the age of our participants, from 45 to 75 years. Heltberg et al. performed a multivariate causal study that concluded that being aged 40–65 years was related to a higher probability of not fulfilling DM care objectives [76]. Similarly, not acting on social-type external factors, like lack of space to facilitate acquiring conducts to promote health, could have an impact [77].

### 4.1. Strengths and Limitations

The simultaneous inclusion of individual, group and community (multicomponent intervention) components to deal with several life habits make this study innovative in the health-promotion field with DM patients.

The fact that barely any evidence exists for the effectiveness of such interventions with DM might be due to the complexity involved in developing and implanting them. It is true that interventions are habitually made with DM on different life habits simultaneously, but prescribing community resources is usually not contemplated. By including this component in our intervention, we evidence its effectiveness in attending to DM patients. We also stress the large number of DM patients who were analyzed, more than expected, as explained by the high DM prevalence for people with two risk factors or more [1,2].

In order to ensure the study’s internal validity, systematized notebooks were used to collect data. Health care professionals were trained in motivational interviews and the intervention’s different components. Group activities were organized with the same structure as with all the PHCCs, and community resources were mapped following the same methodology [39]. However, as the participants and professionals performing the EIRA intervention were not blinded, all the participants could have changed their conduct because they knew that they were being observed.

The flow diagram includes information about the number of participants who dropped out of the study in each phase and their reasons for doing so (see File S2 Table S2). Final losses were 22.19%, which did not exceed the initially foreseen 30%, and losses were practically the same for both groups. High losses during EIRA intervention are a commonplace problem in such studies, and attempts are made to minimize them by reference professionals following up on each participant and including an external support unit to collect data.

In order to foresee this inconvenience, analyses were conducted following the ITT principle, according to which all the subjects randomly assigned to any experimental conditions are included in the analyses, regardless of them not adhering well to the intervention, having dropped out of the study or for any other circumstance that could have taken place after randomization.

The literature also warns that initial imbalances and confounding biases can arise in cluster analyses. We considered this aspect, which led to the multilevel mixed model analysis and the inclusion of auxiliary variables in the imputation, and this was why random forest imputation was followed to avoid collinearity.

The analysis of the baseline values showed no major differences between both groups. It was estimated that for properly performed simple randomization, the probability of a factor showing a 5% statistically significant imbalance would be 5% regardless of the sample size. Therefore, comparisons made using statistical testing are not recommended in the CONSORT guide [34]. According to this guide, the decision to adjust must not be determined by the initial differences being statistically significant, but rather by the previous hypothesis of their strong influence on the result.

BMI or psychosocial support could have been unknown confounding factors, or were not included in the statistical analysis, and could have influenced the effectiveness of the intervention for DM patients. However, other variables, such as ethnic origin or DM duration/seriousness, were not included because EIRA does not specifically address DM patients.

Implementation was complex because it meant having to change work routines that have been ongoing for years. This led to certain organizational problems, which could explain the poor adherence to group and community activities. Moreover, 61% of community activities were not free.

Thus, despite this EIRA intervention being designed as “multicomponent”, according to the participation results we can state that it was carried out mostly by individual and group approaches. This could have been facilitated by the health centers in which PC in Spain is organized, which are physical spaces where a relatively large number of professionals work together and attend to the general public in their area. The disadvantage lies in innovative initiatives not being well accepted by a sufficiently large part of the team when the probability of their implementation not offering guarantees or being of worse quality is higher.

Another main limitation is the need to individualize the EIRA intervention according to DM patients’ intrinsic and extrinsic characteristics [1,2]. Our study took this aspect into account, but its more general characteristics (components, duration, intensity) were standardized to be able to analyze health outcomes, which were obtained as homogeneously and reproducibly as possible. Moreover, the EIRA intervention does not specifically address DM patients, which is why this article included a secondary analysis of a population subgroup.

### 4.2. Implications for Research and Practice

Our study’s external validity is backed by the origin of the patients included in it, who belonged to 26 health centers from all over Spain and different social settings.

Multicomponent interventions that address several unhealthy life habits still need to be further investigated to evidence the effectiveness of this non-pharmacological approach to improve glycemic control, adhere to the Mediterranean diet, practice physical exercise, stop smoking and increase quality of life with DM. This study stresses the need to involve PC in research to improve setting up interventions to promote health.

Regarding future lines of study, physical activity and sedentary lifestyle measures should be refined given their impact on health. The fact that no international consensus exists about how to measure physical activity in PC is stressed, and although several validated questionnaires exist with different characteristics and their strengths and limitations, further evidence is lacking to definitively recommend some more than others [78].

Our study, like other similar ones, analyzes interventions about life habits. Above all, these studies basically evidence the need for organizational changes and their routine inclusion in PC as an offer of integrated services with the community’s co-participation, where implementation strategies will be absolutely necessary.

Set work dynamics and organizations, which are barely flexible to organizational changes, make their generalized implementation difficult. Proof of marked effectiveness for these types of interventions would support them being applied to clinical practice. It is important to point out that the EIRA study marked a before and an after in the participating health centers. Carrying out this innovative research was well accepted by many professionals and health managers, which facilitated its implementation in the usual practice of group and community activities in many health centers where, until that time, it had not been offered.

It is also worth highlighting that to successfully perform multicomponent interventions, it is necessary to seek alliances with other community stakeholders. Even though extending the network of community support is a strong point for non-pharmacological DM treatment, it poses many organizational difficulties that need to be consolidated in the long term.

As for evaluating such interventions, interest shown in the methods set out in the public health field is ever-growing. Recently, Cassetti and Paredes-Carbonell proposed a new conceptual model to explain how these interventions work, where their different components must be identified and what the interaction between each one is like, especially if we consider all the external factors that can influence its development [79].

## 5. Conclusions

The EIRA intervention carried out to promote health by adopting healthy life habits did not significantly improve DM patients’ glycemic control.

Regarding life habits, this intervention significantly improved DM patients adhering to Mediterranean diet but did not obtain significant results for increasing physical activity or for reducing smoking.

DM patients’ quality of life did not significantly change with the EIRA intervention.

Such innovative interventions need further investigation to demonstrate their more long-term effectiveness, also in specific population groups. Moreover, they need implementation strategies from health administrations/organizations to putting them into clinical and community practice, including the analysis of their applicability to different contexts and population groups in PC.

## Figures and Tables

**Table 1:** Baseline characteristics of the patients with diabetes mellitus.

Variables	Control (*n* = 356)		Intervention (*n* = 338)		*p*
	*n*	med. (IQR)/% (95% CI) *	*n*	med. (IQR)/% (95% CI) *)	
**Age**	356	60.00 (54.00–66.00)	338	60.00 (54.00–67.00)	0.901
**Gender** *	356		338		1.000
Male	222	62.36 (57.24–67.28)	210	62.13 (56.87–67.18)	
Female	134	37.64 (32.72–42.76)	128	37.87 (32.82–43.13)	
**Civil status** *	354		335		0.296
Single	48	13.56 (10.29–17.42)	31	9.25 (6.50–12.71)	
Married/Live with a partner	244	68.93 (63.97–73.58)	238	71.04 (66.02–75.71)	
Separated and/or divorced	36	10.17 (7.35–13.64)	43	12.84 (9.58–16.73)	
Widow/Widower	25	7.06 (4.74–10.08)	23	6.87 (4.52–9.95)	
Other	1	0.28 (0.03–1.31)	0	0.00 (0.00–0.00)	
**Country born in** *	355		335		0.420
Spain	326	91.83 (88.64–94.34)	318	94.93 (92.18–96.90)	
Rest of Europe	8	2.25 (1.07–4.21)	5	1.49 (0.57–3.24)	
America	17	4.79 (2.92–7.39)	9	2.69 (1.34–4.85)	
Asia	0	0.00 (0.00–0.00)	0	0.00 (0.00–0.00)	
Africa	4	1.13 (0.38–2.66)	3	0.90 (0.25–2.37)	
**Spanish Autonomous Community** *	356		338		0.000
Andalusia	52	14.61 (11.23–18.56)	48	14.20 (10.79–18.22)	
Aragón	29	8.15 (5.64–11.33)	56	16.57 (12.90–20.81)	
Balearics	79	22.19 (18.11–26.72)	72	21.30 (17.19–25.90)	
Castilla y León	33	9.27 (6.59–12.61)	49	14.50 (11.05–18.55)	
Catalonia	49	13.76 (10.48–17.63)	43	12.72 (9.49–16.59)	
Galicia	59	16.57 (12.99–20.70)	46	13.61 (10.27–17.57)	
Basque Country	55	15.45 (11.98–19.48)	24	7.10 (4.71–10.21)	
**Level of education** *	353		335		0.882
Higher Education	42	11.90 (8.83–15.58)	39	11.64 (8.54–15.40)	
Secondary Education	123	34.84 (30.01–39.92)	118	35.22 (30.25–40.45)	
Primary Education	157	44.48 (39.35–49.69)	154	45.97 (40.69–51.32)	
No studies	31	8.78 (6.16–12.07)	24	7.16 (4.77–10.30)	
**Occupation** *	355		335		0.746
Student	0	0.00 (0.00–0.00)	1	0.30 (0.03–1.39)	
Employee	91	25.63 (21.30–30.36)	83	24.78 (20.38–29.60)	
Self-employed	37	10.42 (7.57–13.92)	34	10.15 (7.26–13.72)	
Leave from work >3 months	8	2.25 (1.07–4.21)	11	3.28 (1.76–5.61)	
Unemployed and paid	24	6.76 (4.49–9.73)	14	4.18 (2.41–6.73)	
Unemployed not paid	14	3.94 (2.28–6.35)	14	4.18 (2.41–6.73)	
Household tasks	37	10.42 (7.57–13.92)	41	12.24 (9.06–16.07)	
Permanent disability	17	4.79 (2.92–7.39)	12	3.58 (1.97–5.99)	
Retired	127	35.77 (30.92–40.86)	125	37.31 (32.26–42.58)	
**Glycemic control**					
HbA1c (%)	279	6.80 (6.30–7.90)	253	6.90 (6.40–7.70)	0.884
Regular/good control (HbA1c <8%) (*Yes*) *	215	77.06 (71.87–81.70)	205	81.03 (75.86–85.49)	0.322
**Mediterranean diet**					
Adhering to Mediterranean diet (MEDAS)	354	7.00 (5.00–8.00)	338	7.00 (5.00–8.00)	0.828
Good adherence (MEDAS ≥9) (*Yes*) *	73	20.62 (16.66–25.06)	55	16.27 (12.63–20.49)	0.238
Diet quality (DQI-I)	354	38.00 (36.00–41.00)	335	38.00 (36.00–40.00)	0.576
**Physical activity**					
Little physical activity (IPAQ)	353		328		0.818
Moderate/Intensive *	168	47.59 (42.42–52.80)	153	46.65 (41.30–52.05)	
Low (<600 MET.min/week) *	185	52.41 (47.20–57.58)	175	53.35 (47.95–58.70)	
Sedentary lifestyle (≥6 h/week sat down) (*Yes*) *	111	42.05 (36.20–48.06)	98	38.13 (32.35–44.18)	0.373
**Smoking habit**					
≥1 cigarette/day (*Yes*) *	145	40.73 (35.72–45.89)	127	37.57 (32.53–42.83)	0.437
**Quality of life**					
VAS (EuroQol-5D5L)	348	70.00 (50.00–80.00)	335	70.00 (50.00–80.00)	0.676

*n*: absolute value; med.: median; IQR: interquartile range; %: percentage; CI: confidence interval; HbA1c: glycosylated hemoglobin; MEDAS: Mediterranean diet adherence questionnaire; DQI-I: international diet quality index; IPAQ: international physical activity questionnaire; VAS: visual analogue scale of the EuroQol-5D5L quality of life questionnaire; P: statistical significance. * expressed as % (95% CI).

**Table 2:** Descriptive postintervention analysis of the outcome variables.

Variables	Control (*n* = 356)		Intervention (*n* = 338)	
	*n*	med. (IQR)/% (95% CI) *	*n*	med. (IQR)/% (95% CI) *
**Glycemic control**				
HbA1c (%)	188	6.80 (6.30–7.70)	169	6.70 (6.20–7.40)
Regular/good control (HbA1c <8%) (*Yes*) *	153	81.38 (75.36–86.45)	152	89.94 (84.73–93.80)
**Mediterranean diet**				
Adhering to Mediterranean diet (MEDAS)	268	8.00 (6.00–9.00)	262	8.00 (7.00–9.00)
Good adherence (MEDAS ≥9) (*Yes*) *	88	32.84 (27.42–38.62)	106	40.46 (34.65–46.48)
Diet quality (DQI-I)	259	39.00 (37.00–41.00)	246	39.00 (37.00–41.00)
**Physical activity**				
Little physical activity (IPAQ)	256		250	
Moderate/Intensive *	138	52.08 (46.07–58.04)	137	52.29 (46.24–58.29)
Low (<600 MET.min/week) *	127	47.92 (41.96–53.93)	175	47.71 (46.71–53.76)
Sedentary lifestyle (≥6 h/week sat down) (*Yes*) *	149	43.57 (38.38–48.86)	138	42.86 (37.54–48.31)
**Smoking habit**				
≥1 cigarette/day (*Yes*) *	103	38.85 (32.89–44.51)	85	32.57 (27.10–38.42)
**Quality of life**				
VAS (EuroQol-5D5L)	261	70.00 (60.00–80.00)	260	70.00 (60.00–80.00)

*n*: absolute value; med.: median; IQR: interquartile range; %: percentage; CI: confidence interval; HbA1c: glycosylated hemoglobin; MEDAS: Mediterranean diet adherence questionnaire; DQI-I: international diet quality index; IPAQ: international physical activity questionnaire; VAS: visual analogue scale of the EuroQol-5D5L quality of life questionnaire. * expressed as % (95% CI); P: statistical significance.

**Table 3:** Adjusted effectiveness of the intervention in patients with DM. Results by ITT (*n* = 694).

Variables	Adjusted Mean Difference	OR	95% CI	*p*	
HbA1c (%)	−0.09		−0.29–0.10	0.327	NS
Regular/good glycemia control (*Yes*)		0.57	0.25–1.31	0.170	NS
Diet quality (DQI-I)	0.25		−0.32–0.82	0.392	NS
Adhering to Mediterranean diet (MEDAS)	0.45		0.01–0.89	0.043	*
Good adherence to Mediterranean diet (*Yes*)		1.62	1.03–2.54	0.036	*
Moderate/intensive physical activity (*Yes*)		1.09	0.64–1.86	0.740	NS
Sedentary lifestyle (*No*)		0.97	0.55–1.73	0.922	NS
Smoke ≥1 cigarette/day (*No*)		0.61	0.54–1.06	0.079	NS
VAS (EuroQol-5D5L)	−1.26		−4.98–2.45	0.504	NS

NS: *p* > 0.05; * *p* < 0.05. CI: confidence interval; HbA1c: glycosylated hemoglobin; %: percentage; MEDAS: questionnaire of adherence to the Mediterranean diet; DQI-I: international diet quality index; VAS: scale visual analogue of the EuroQol-5D5L quality of life questionnaire; P: statistical significance.

## Data Availability

The data that support the findings of this study are available on reasonable request from the corresponding author at franciscorepresascarrera@gmail.com. Proposals requesting data access will need to specify how it is planned to use the data.

## Supplementary Materials

The following are available online at https://www.mdpi.com/article/10.3390/ijerph18115788/s1. File S1: Independent variables, File S2: Primary healthcare centers, recruitment and follow-up of the protocol and adjusted effectiveness of the intervention for complete cases, File S3: Lifestyle, clinical values, pharmacological treatment, comorbidity, quality of life and psychosocial evaluation.

## Nomenclature

%	Percentage
ABI	Ankle-Brachial Index
APP	Mobile application
BMI	Body Mass Index
CAVI	Heart-Ankle Vascular Index
CG	Control Group
C-HDL	High-Density Lipoprotein Cholesterol
CI	Confidence Interval
C-LDLCONSORT	Low-Density Lipoprotein CholesterolConsolidated Standards of Reporting Trials
COPD	Chronic Obstructive Pulmonary Disease
CT	Total Cholesterol
CVD	Cerebrovascular Disease
DBP	Diastolic Blood Pressure
DM	Diabetes Mellitus
DQI	Diet Quality
DQI-I	International Diet Quality Index
GAD-7	Generalized Anxiety Disorder-7
HbA1c	Glycosylated hemoglobin
HTA	Arterial Hypertension
IG	Intervention Group
IPAQ	International Physical Activity Questionnaire
IQR	Interquartile Range
ITT	Intention To Treat
med.	Median
MEDAS	Mediterranean Diet Adherence Questionnaire
n	Absolute value
VAS	Visual Analogue Scale
OR	Odds Ratio
P	Statistical significance
PC	Primary Care
PHCCs	Primary Health Care Centers
RCT	Randomized Clinical Trial
RedIAPP	Research Network in Preventive and Health Promotion Activities
SBP	Systolic blood pressure
SMS	Text messages
