# Supplementary material for: Effectiveness of a Multicomponent Intervention in Primary Care That Addresses Patients with Diabetes Mellitus with Two or More Unhealthy Habits, Such as Diet, Physical Activity or Smoking: Multicenter Randomized Cluster Trial (EIRA Study)
Source: Int J Environ Res Public Health. 2021 May 28;18(11):5788. doi: 10.3390/ijerph18115788 (PMC8198299; doi:10.3390/ijerph18115788)
Supplement: Supplementary file 1 [file ijerph-18-05788-s001.zip › ijerph-1171659-supplementary/File S1.pdf]

APPENDIX 1:

Table S1. Independent variables

Primary Care Centres (PHCCs): teaching unit [yes], population served [N], the population's age [years], professionals' age [years], experience of professionals in primary care [years], doctors [N] , medical quota [N], average number of medical visits per day [N], average time per medical visit [minutes], nurses [N], nursing quota [N], average number of nurse visits per day [N], average time per visit per nurse [minutes], social workers [N], and average time per visit per social worker [minutes].

Quality of life: The EuroQol-5D5L questionnaire including the EuroQol-5D5L index and health status 12 months ago [better/same/worse] [1].

Motivation for behaviour change: precontemplative, contemplative, preparation, action and maintenance [2].

Clinical values: body mass index (BMI) [kg/m^2], abdominal circumference [cm], glucose [mg/dl], total cholesterol [mg/dl], HDL cholesterol (C-HDL) [mg/dl], LDL cholesterol (C-LDL) [mg/dl], triglycerides [mg/dl], systolic blood pressure (SBP) [mmHg], diastolic blood pressure (DBP) [mmHg], ankle-brachial index (ABI) right and left, heart-ankle vascular index (CAVI) right and left, nicotine dependence (Fageström) [3] and cooximetry [ppm].

Pharmacological treatment: antiplatelet agents, oral antidiabetics, insulin, antihypertensive drugs, and lipid-lowering drugs.

Comorbidity: smoking, obesity, dyslipidaemia, mixed dyslipidaemia, hypercholesterolaemia, hypertriglyceridaemia, arterial hypertension (HTA), peripheral vascular disease, cerebrovascular disease (CVD), heart disease, kidney disease, haemiplegia, peptic ulcer, connective tissue disease, and chronic obstructive pulmonary disease (COPD).

Psychosocial: We evaluated the social support to the DUKE-UNC-11 questionnaire [4] and the generalised anxiety disorder with the GAD-7 questionnaire [5]. Depression was assessed by the PHQ-9 questionnaire [6] and the CIDI composite international diagnostic interview [7]. Psychiatric symptoms were evaluated by the HSCL-25 questionnaire [anxiety/depression] [8].

1. EuroQol-5D-5L Badia X, Roset M, Montserrat S, Herdman M, Segura A. The Spanish version of EuroQol: description and applications. Med Clin (Barc) 1999; 112 (Supl 1): 79-86.
2. Prochaska JO, Redding CA, Evers KE. The Transtheoretical model and stages of change. In: Glanz K, Rimer BK, Viswanath K, editors. Health behavior and health education. Theory, research, and practice. 4th ed. San Francisco, CA: John Wiley & Sons, Inc; 2008. p. 97–122.
3. Becoña E, Vázquez FL. The Fagerström Test for Nicotine Dependence in a Spanish sample. Psychol Rep. 1998 Dec;83(3 Pt 2):1455-8. DOI: 10.2466/pr0.1998.83.3f.1455
4. Bellón JA, Delgado A, Luna J, Lardelli P. Validity and reliability of the Duke-UNC-11 functional social support questionnaire. Aten Primaria, 1996; 18: 153-63.
5. García-Campayo et al. Cultural adaptation into Spanish of the generalized anxiety disorder-7 (GAD-7) scale as a screening tool. Health and Quality of Life Outcomes 2010;8:8. DOI: 10.1186/1477-7525-8-8
6. Spitzer RL, Kroenke K, Williams JB. Patient Health Questionnaire Primary Care Study Group. Validation and utility of a self-report version of the PRIME-MD: the PHQ primary care study. JAMA. 1999;282:1737–44. DOI: 10.1001/jama.282.18.1737
7. Rubio-Stipec M, Bravo M, Canino G. La Entrevista Diagnóstica Internacional Compuesta (CIDI): Un instrumento epidemiológico adecuado para ser administrado conjuntamente con otros sistemas diagnósticos en diferentes culturas. Acta Psiquiátrica Psicológica de América Latina 1991; 37: 191-204
8. Sandanger I, Moum T, Ingebrigtsen G, Dalgard OS, Sørensen T, Bruusgaard D. Concordance between symptom screening and diagnostic procedure: the Hopkins Symptom Checklist-25 and the Composite International Diagnostic Interview I. Soc Psychiatry Psychiatr Epidemiol 1998;33(7):345-354. DOI: 10.1007/s001270050064
