# Supplementary material for: Effectiveness of a Multicomponent Intervention in Primary Care That Addresses Patients with Diabetes Mellitus with Two or More Unhealthy Habits, Such as Diet, Physical Activity or Smoking: Multicenter Randomized Cluster Trial (EIRA Study)
Source: Int J Environ Res Public Health. 2021 May 28;18(11):5788. doi: 10.3390/ijerph18115788 (PMC8198299; doi:10.3390/ijerph18115788)
Supplement: Supplementary file 1 [file ijerph-18-05788-s001.zip › ijerph-1171659-supplementary/File S2.pdf]

APPENDIX 2:

Table S1. Characteristics of the Primary Healthcare Centres (PHCCs)

Variables	Control PHCC (N=13)		Intervention PHCC (N=12)	
	N	med. (IQR) / % (95% CI)*	N	med. (IQR) / % (95% CI)*
**Training Unit** *(Yes)* *	11	84.62 (57.77 to 95.67)	8	66.67 (39.06 to 86.19)
**Population served** (N)		16.054 (13.819 to 22.487)		22.482 (17.573 to 26.930)
Age (years)		45.25 (40.54 to 48.58)		42.73 (38.83 to 44.35)
**Professionals**				
Age (years)		49.80 (47.50 to 53.80)		50.50 (46.40 to 53.80)
Experience PC (years)		18.10 (15.10 to 24.30)		18.80 (17.10 to 20.80)
**Doctors** (N)		12.00 (9.00 to 15.00)		13.00 (8.00 to 14.00)
Quota		1.467 (1.400 to 1.600)		1.576 (1.442 to 1.708)
Mean visits/day		32.20 (34.38 to 25.90)		30.00 (27.00 to 37.79)
Mean visiting time (minutes)		7.00 (7.00 to 8.00)		7.00 (7.00 to 8.00)
**Nurses** (N)		10.00 (7.00 to 17.00)		10.00 (7.00 to 15.00)
Quota		1.450 (1.218 to 1.467)		1.555 (1.430 to 1.896)
Mean visits/day		18.80 (4.00 to 12.00)		15.00 (12.00 to 20.10)
Mean visiting time (minutes)		10.00 (9.40 to 15.00)		12.40 (10.00 to 15.00)
**Social Workers** (N)		1.00 (1.00 to 1.00)		1.00 (1.00 to 1.00)
Mean visiting time (minutes)		18.20 (15.00 to 20.00)		20.00 (15.00 to 20.00)

N: absolute value / med.: median / IQR: interquartile range / %: percentage / CI: confidence interval / PC: primary care / PHCC: primary health care centre.
*expressed as % (95%CI)

Table S2. Recruitment and follow-up of the protocol for patients with Diabetes Mellitus

Variables	Control (N=356)		Intervention (N=338)	
	N	% (95% CI) / med. (IQR)*	N	% (95% CI) / med. (IQR)*
**Recruitment**	356	100.00 (100.00 to 100.00)	338	100.00 (100.00 to 100.00)
Poorly adhering to Mediterranean diet	325	91.29 (88.03 to 93.89)	317	93.79 (90.83 to 95.99)
Little physical activity	327	92.85 (88.67 to 94.67)	312	92.31 (89.10 to 94.79)
Smoking	145	40.73 (35.72 to 45.89)	127	36.69 (31.68 to 41.92)
Risky lifestyles found				
N=2	271	76.12 (71.50 to 80.33)	261	77.22 (72.53 to 81.45)
N=3	85	23.88 (19.67 to 28.50)	77	22.78 (18.55 to 27.47)
**Basal evaluation**	356	100.00 (100.00 to 100.00)	338	100.00 (100.00 to 100.00)
**First intervention visit**			307	90.83 (87.40 to 93.56)
**Starting intervention**				
Mediterranean diet			286	90.22 (86.45 to 93.02)
Physical activity			281	90.06 (86.24 to 92.91)
Smoking			114	91.93 (85.79 to 95.56)
**Undertaking intervention**				
Individual follow-up visits *			338	1.00 (0.00 to 2.00)
Using text messages service			201	59.47 (54.17 to 64.60)
Using the web platform			14	12.39 (7.28 to 19.39)
Using APPs			5	1.63 (0.62 to 3.53)
Mediterranean diet				
Delivering written material			181	53.55 (48.22 to 58.82)
Deriving in group activity			67	19.82 (15.84 to 24.32)
Deriving in community activity			25	13.81 (0.95 to 19.59)
Physical activity				
Delivering written material			193	57.10 (51.78 to 62.30)
Deriving in group activity			14	4.14 (2.39 to 6.67)
Deriving in community activity			44	22.80 (17.44 to 29.21)
Smoking				
Delivering written material			1	0.88 (0.16 to 4.80)
Deriving in group activity			10	8.77 (4.83 to 15.40)
Deriving in community activity			3	2.63 (0.90 to 7.46)
**Final evaluation (12 months)**	277	77.81 (73.28 to 81.89)	263	77.81 (73.16 to 81.99)
Mediterranean diet	253	77.85 (73.02 to 82.02)	247	77.92 (73.03 to 82.13)
Physical activity	254	77.68 (72.86 to 81.85)	243	77.88 (72.96 to 82.14)
Smoking	110	75.86 (68.29 to 82.10)	99	79.84 (71.93 to 85.95)
**Leaving the study**	79	22.19 (18.18 to 26.79)	75	22.19 (18.09 to 26.92)
For no known reason	19	5.34 (3.44 to 8.18)	26	7.69 (5.30 to 11.03)
Not attending the final evaluation	31	8.71 (6.20 to 12.10)	24	7.10 (4.82 to 10.35)
Removed their consent	16	4.49 (2.79 to 7.18)	17	5.03 (3.16 to 7.91)
Died	0	0.00 (0.00 to 0.00)	0	0.00 (0.00 to 0.00)
Personal reasons	13	3.65 (2.15 to 6.15)	8	2.37 (1.20 to 4.60)

N: absolute value / med.: median / IQR: interquartile range / %: percentage / CI: confidence interval.
*expressed as med. (IQR)

Table S3. Adjusted effectiveness of the intervention in patients with DM. Results for complete cases (N=694)

Variables	Adjusted mean difference	OR	95% CI	*p*
HbA1c (%)	-0.10		-0.28 to 0.09	NS
Regular/good glycaemia control *(Yes)*		0.37	0.16 to 0.85	*
Diet quality (DQI-I)	0.24		-0.36 to 0.86	NS
Adhering to Mediterranean diet (MEDAS)	0.54		0.07 to 1.02	*
Good adherence to Mediterranean diet *(Yes)*		1.78	1.02 to 3.15	*
Moderate/intensive physical activity *(Yes)*		1.18	0.64 to 2.30	NS
Sedentary lifestyle *(No)*		0.97	0.49 to 1.89	NS
Smoke ≥1 cigarette/day *(No)*		0.41	0.19 to 0.82	*
VAS (EuroQol-5D5L)	-1.85		-6.23 to 2.50	NS

OR: odds ratio / CI: confidence interval / HbA1c: glycosylated haemoglobin / %: percentage / MEDAS: questionnaire of adherence to the Mediterranean diet / DQI-I: international diet quality index / VAS: scale visual analogue of the EuroQol-5D5L quality of life questionnaire / *p*: statistical significance.

NS: $p > 0.05$ *$p < 0.05$
