# Supplementary material for: Effectiveness of a Multicomponent Intervention in Primary Care That Addresses Patients with Diabetes Mellitus with Two or More Unhealthy Habits, Such as Diet, Physical Activity or Smoking: Multicenter Randomized Cluster Trial (EIRA Study)
Source: Int J Environ Res Public Health. 2021 May 28;18(11):5788. doi: 10.3390/ijerph18115788 (PMC8198299; doi:10.3390/ijerph18115788)
Supplement: Supplementary file 1 [file ijerph-18-05788-s001.zip › ijerph-1171659-supplementary/File S3.pdf]

APPENDIX 3:

Table S1. Reason for lifestyle-related change of conduct of patients with Diabetes Mellitus

Variables	PRE-INTERVENTION			
	Control (N=356)		Intervention (N=338)	
	N	% (95% CI)	N	% (95% CI)
**Mediterranean diet**	347		292	
Precontemplative	128	36.89 (31.94 to 42.06)	12	4.11 (2.27 to 6.85)
Contemplative	74	21.33 (17.26 to 25.86)	49	16.78 (12.83 to 21.38)
Preparation	47	13.54 (10.25 to 17.44)	151	51.71 (45.99 to 57.40)
Action	37	10.66 (7.74 to 14.24)	60	20.55 (16.22 to 25.46)
Maintenance	61	17.58 (13.85 to 21.84)	20	6.85 (4.37 to 10.18)
**Physical activity**	349		316	
Precontemplative	87	24.93 (20.61 to 29.66)	30	9.49 (6.63 to 13.09)
Contemplative	35	10.03 (7.21 to 13.51)	31	9.81 (6.90 to 13.46)
Preparation	12	3.44 (1.89 to 5.75)	34	10.76 (7.70 to 14.53)
Action	3	0.86 (0.24 to 2.28)	9	2.85 (1.42 to 5.13)
Maintenance	212	60.74 (55.55 to 65.76)	212	67.09 (61.77 to 72.10)
**Smoking**	346		292	
Precontemplative	139	40.17 (35.11 to 45.40)	30	10.27 (7.18 to 14.15)
Contemplative	98	28.32 (23.77 to 33.24)	59	20.21 (15.91 to 25.09)
Preparation	55	15.90 (12.33 to 20.03)	114	39.04 (33.58 to 44.72)
Action	28	8.09 (5.56 to 11.32)	64	21.92 (17.46 to 26.92)
Maintenance	26	7.51 (5.09 to 10.65)	25	8.56 (5.76 to 12.18)

N: absolute value / %: percentage / CI: confidence interval

Table S2. Clinical values and pharmacological treatment of patients with Diabetes Mellitus

Variables	PRE-INTERVENTION			
	Control (N=356)		Intervention (N=338)	
	N	med. (IQR)	N	med. (IQR)
BMI (kg/m^2)	355	31.14 (27.50 to 34.4)	332	31.98 (28.39 to 36.14)
Abdominal girth (cm)	346	106.15 (97.50 to 116.00)	325	107.00 (99.00 to 117.00)
Glucose (mg/dl)	342	128.00 (109.00 to 150.00)	325	132.00 (112.00 to 152.00)
Total cholesterol (mg/dl)	344	183.00 (158.50 to 213.50)	326	180.00 (157.00 to 209.00)
C-HDL (mg/dl)	335	45.00 (37.00 to 54.00)	314	45.00 (38.00 to 53.00)
C-LDL (mg/dl)	328	107.00 (87.10 to 132.00)	306	103.00 (80.00 to 131.00)
Triglycerides (mg/dl)	343	142.00 (102.00 to 196.00)	325	142.00 (103.30 to 191.00)
SBP (mmHg)	355	135.00 (125.00 to 145.00)	331	136.00 (125.00 to 146.00)
DBP (mmHg)	355	80.00 (73.00 to 88.00)	330	82.00 (77.00 to 88.00)
Right ABI	202	1.08 (0.98 to 1.16)	213	1.05 (0.95 to 1.13)
Left ABI	202	1.09 (0.98 to 1.15)	214	1.05 (0.92 to 1.13)
Right CAVI	200	8.30 (7.50 to 9.25)	213	8.70 (7.50 to 9.40)
Left CAVI	201	8.50 (7.50 to 9.30)	213	8.60 (7.50 to 9.50)
Nicotine dependence (Fageström)	122	3.00 (1.00 to 3.00)	105	3.00 (1.00 to 4.00)
Cooximetry (ppm)	89	9.00 (0.00 to 19.00)	91	8.00 (2.00 to 16.00)
**Pharmacological Treatment**	**N**	**% (95% CI)**	**N**	**% (95% CI)**
Antiplatelet	77	21.94 (17.85 to 26.49)	85	25.76 (21.27 to 30.67)
Oral antidiabetics	269	76.20 (71.56 to 80.42)	230	69.70 (64.58 to 74.47)
Insulin	77	21.88 (17.80 to 26.41)	41	12.42 (9.20 to 16.31)
Antihypertensives	219	62.04 (56.90 to 66.99)	214	64.85 (59.59 to 69.85)
Hypolipidemics	208	58.92 (53.74 to 63.97)	209	62.95 (57.66 to 68.02)

N: absolute value / med.: median / IQR: interquartile range / %: percentage / CI: confidence interval / BMI: body mass index / C-HDL: high-density lipoprotein cholesterol / C-LDL: low-density lipoprotein cholesterol / SBP: systolic blood pressure / DBP: diastolic blood pressure / ABI: ankle-brachial index / CAVI: heart-ankle vascular index.

Table S3. Comorbidity of the patients with Diabetes Mellitus

Variables	Control (N=356)		Intervention (N=338)	
	N	% (95% CI)	N	% (95% CI)
Smoking	145	40.73 (35.72 to 45.89)	127	37.57 (32.53 to 42.83)
Obesity	202	56.90 (51.71 to 61.98)	213	64.16 (58.89 to 69.17)
Dyslipidemia	315	90.52 (87.10 to 93.26)	302	92.92 (89.75 to 95.34)
Mixed Dyslipidemia	218	63.56 (56.37 to 68.52)	186	57.23 (51.81 to 62.53)
Hypercholesterolemia	171	50.89 (45.56 to 56.21)	150	47.62 (42.15 to 53.14)
Hypertriglyceridemia	159	46.36 (41.13 to 51.65)	148	45.54 (40.18 to 50.97)
HTA	245	69.21 (64.26 to 73.85)	230	69.28 (64.16 to 74.06)
Peripheral Vascular Disease	15	4.24 (2.50 to 6.72)	20	5.98 (3.81 to 8.92)
CVD	13	3.67 (2.07 to 6.02)	18	5.39 (3.34 to 8.20)
Cardiopathy	34	9.63 (6.88 to 13.04)	37	11.08 (8.05 to 14.78)
Cancer	22	6.21 (4.05 to 9.09)	18	5.37 (3.33 to 8.18)
Hepatopathy	12	3.39 (1.87 to 5.67)	16	4.77 (2.87 to 7.46)
Kidney Disease	10	2.82 (1.46 to 4.95)	6	1.79 (0.75 to 3.65)
Hemiplegia	1	0.28 (0.03 to 1.31)	0	0 (0.00 to 0.00)
Peptic Ulcer	1	0.28 (0.03 to 1.31)	2	0.59 (0.12 to 1.90)
Connective Tissue Disease	12	3.39 (1.87 to 5.67)	9	2.68 (1.34 to 4.85)
COPD	21	5.93 (3.82 to 8.76)	22	6.57 (4.28 to 9.60)

N: absolute value / %: percentage / CI: confidence interval / HTA: arterial hypertension / CVD: cerebrovascular disease / COPD: chronic obstructive pulmonary disease

Table S4. Quality of life of the patients with Diabetes Mellitus

Variables	PRE-INTERVENTION			
	Control (N=356)		Intervention (N=338)	
	N	% (95% CI) / med. (IQR)*	N	% (95% CI) / med. (IQR)*
EuroQol-5D5L Index *	354	0.79 (0.70 to 1.00)	335	0.80 (0.74 to 1.00)
Health status 12 months ago	353		328	
Better	85	24.08 (19.84 to 28.74)	72	21.95 (17.73 to 26.66)
Same	197	55.81 (50.60 to 60.92)	181	55.18 (49.78 to 60.50)
Worse	71	20.11 (16.19 to 24.53)	75	22.87 (18.57 to 27.64)

N: absolute value / med.: median / IQR: interquartile range / %: percentage / CI: confidence interval.
*expressed as med. (IQR)

Table S5. Psychosocial evaluation of the patients with Diabetes Mellitus

Variables	PRE-INTERVENTION			
	Control (N=356)		Intervention (N=338)	
	N	% (95% CI) / med. (IQR)*	N	% (95% CI) / med. (IQR)*
Social support (DUKE-UNC-11) *	354	48.00 (41.00 to 52.00)	332	47.00 (41.00 to 53.00)
Generalized anxiety (GAD-7) *	349	2.00 (0.00 to 6.00)	331	2.00 (0.00 to 5.00)
Composite International Diagnostic Interview (CIDI)				
Depression	15	5.07 (2.99 to 8.01)	14	4.61 (2.66 to 7.40)
Depression questionnaire (PHQ-9) *	346	2.50 (1.00 to 7.00)	334	3.00 (1.00 to 6.00)
Depression	18	5.20 (3.22 to 7.92)	17	5.09 (3.11 to 7.84)
Psychiatric symptoms (HSCL-25)				
Anxiety	27	36.49 (26.20 to 47.80)	57	42.86 (34.68 to 51.35)
Depression	16	21.62 (13.44 to 31.97)	45	33.83 (26.21 to 42.16)

N: absolute value / med.: median / IQR: interquartile range / %: percentage / CI: confidence interval / DUKE-UNC-11: Duke questionnaire for functional social support / GAD-7: scale for generalized anxiety disorder / CIDI: composite international diagnostic interview / PHQ-9: health questionnaire of the patient / HSCL-25: scale of psychiatric symptoms Hopkins.
*expressed as med. (IQR)
